# Supplementary material for: How to measure the local Dzyaloshinskii-Moriya Interaction in Skyrmion Thin-Film Multilayers
Source: Sci Rep. 2019 Feb 28;9:3114. doi: 10.1038/s41598-019-39501-x (PMC6395602; doi:10.1038/s41598-019-39501-x)
Supplement: Supplementary file 1 — Supporting material on How to measure the local Dzyaloshinskii‐Moriya Interaction in Skyrmion Thin‐Film Multilayers [file 41598_2019_39501_MOESM1_ESM.pdf]

Supporting material
How to measure the local Dzyaloshinskii-Moriya Interaction in Skyrmion Thin-Film Multilayers

Mirko Baćani,[1] Miguel A. Marioni,[1,*] Johannes Schwenk,[1,2] Hans J. Hug[1,2]

1 Empa, Swiss Federal Laboratories for Materials Science and Technology, CH-8600 Dübendorf, Switzerland

2 Department of Physics, University of Basel, CH-4056 Basel, Switzerland

Average Dzyaloshinskii-Moriya interaction

The domain pattern in thin films results from a competition between the reduction of magnetostatic energy through the formation of magnetic domains and the increase in exchange and anisotropy energies through the corresponding creation of domain wall surface. At stable equilibrium the combined magnetostatic and wall energy is at its minimum. This condition represents a constraint for the both energy contributions that can be written in terms of the domain geometry, and which can then be inverted to obtain the wall energy density σ_w as a function of the geometry. Using

$$\sigma_w = 4\sqrt{A\,K_u} - \pi D \tag{1}$$

we can then extract the DM interaction coefficient D. The fastest practical way to obtain σ_w is to assume we have regular stripe domains at equilibrium. Then we can measure the stripe width experimentally, and obtain D. The problem with this approach is threefold. First, our system may be in a metastable state far from equilibrium, for which the constraint is not applicable. Second, the domain pattern may not consist of a regular pattern of stripe domains, so we will be using an incorrect expression for the constraint between magnetostatic and wall energies. Lastly, we may not be able to correctly estimate the relevant geometrical parameter, the stripe width, say, so we will be using questionable data in our estimate.

To overcome these difficulties but retain the advantage of ascertaining D from a simple MFM measurement, we first demagnetize the system in different ways, e.g. with in- and out-

of-plane oscillatory-decaying fields, thereby ensuring that we are close to the stable minimum energy configuration and that the result is robust. Next, we use the exact domain pattern geometry that we measured. To explore the energy minima, we use the same magnetic domain pattern but (virtually) vary its scale. This guarantees that our minimization can reach the measured pattern, and renders finding a good measure of the pattern geometry at equilibrium a trivial problem.

For an arbitrary pattern of magnetization in zero field (e.g. Figure 1a), henceforth assumed to correspond to a scaling factor s = 1 we calculate the magnetostatic energy of the stack of 6 Co layers (with 653.6 kA/m of magnetization) assuming they extend without distortion through the Co layers. For convenience we carry out the calculation in 2D reciprocal space. We multiply the wall energy (areal) density with the total thickness of all Co layers and with the total length of the domain walls. Then we add the magnetostatic energy calculated for the given domain pattern in the Co films and the Co-film interaction energies, and divide the result by the film area. The wall energy includes the exchange stiffness, which we select within the range of literature values,[1,2] A = 16 pJ/m, the (uniaxial) anisotropy K_u = 0.414 MJ/m^3, and crucially the strength of the DM interaction, D, as a parameter for which initial values can be selected. The process is repeated for a range of scaling factors s. Although the domain wall length scales with s, the domain width is constant, forcing a recalculation of the magnetostatic for each scale. We thus obtain an energy curve, for instance for D = 2.05 mJ/m^2, which is minimal at a scale of ≈ 6.5µm/5µm=1.30 (blue curve, Figure 1b). This means that for the D selected the equilibrium domain pattern would have larger features by a factor of 1.3. We repeat the calculations for a different D, until the minimum is found at scale s = 1.0. The value of D used at that point is the average D for this pattern, e.g. 2.09 mJ/m^2 in Figure 1b.

For comparison, we repeat the process for a pattern of regular stripe domains (Figure 1c) with width matching the average domain width measured from Figure 1a. We arrive at a distinctively lower value of D (1.72 mJ/m^2). This shows the extent of the systematic error in D when we represent the measured domain patterns as regular stripe domains.

For this work we apply this procedure to demagnetized patterns. Independently of the (measured) domain pattern utilized, we obtain similar values of D near 1.97 mJ/m^2. The average of such calculations results in the value for D and a measure of the error, 1.97 ±

0.03 mJ/m^2 for the perpendicularly demagnetized films. Note that once more, the results for strip patterns are significantly lower.

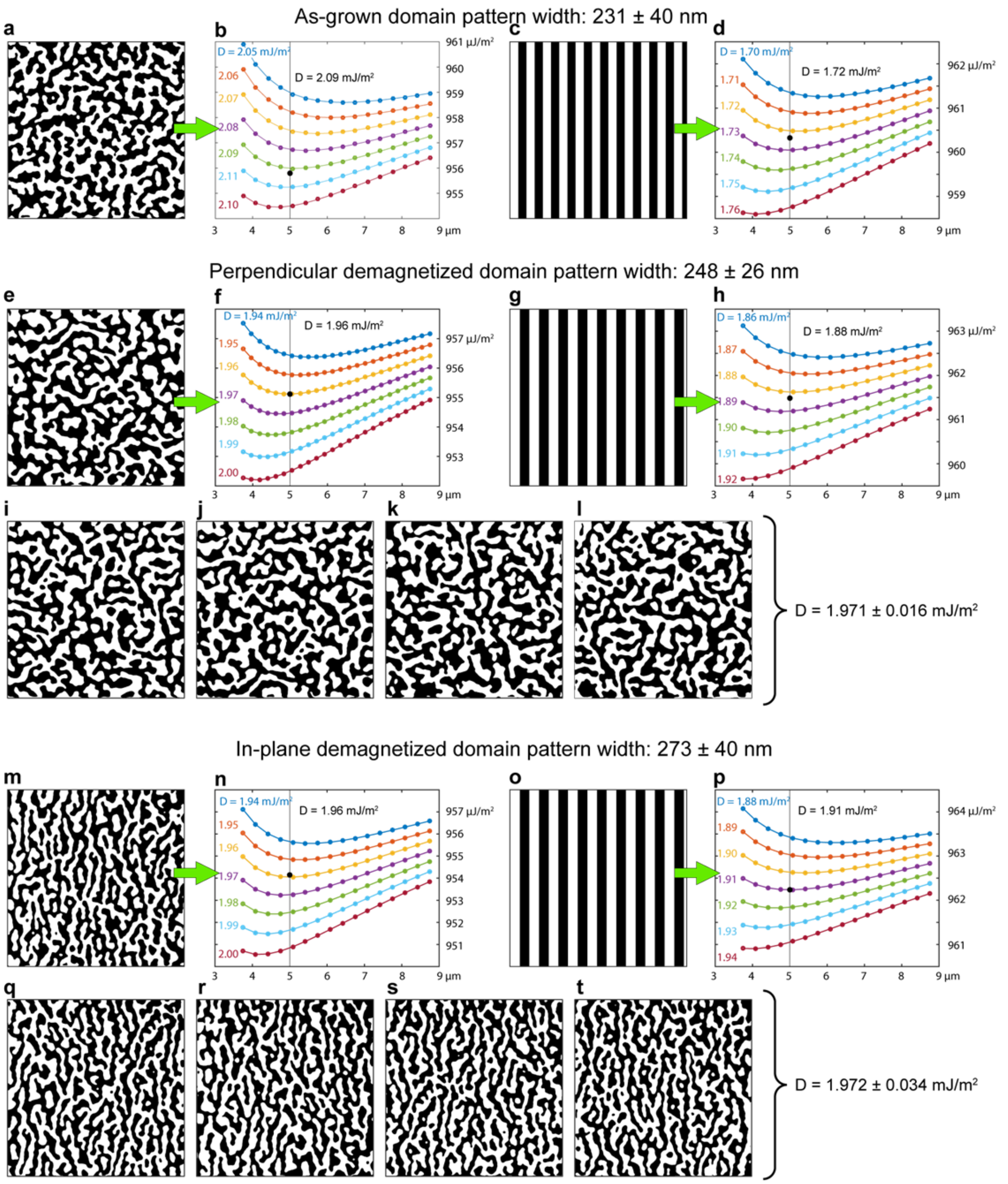

Figure 1: Method for finding the average DM interaction coefficient D. All measured and reference stripe domain patterns cover an area of 5 $\times$ 5 μm^2. **a** As grown domain pattern. **b** Energy density as a function of the assumed scale of the pattern in **a** with parameter D. For a given parameter D, the scale that minimizes the energy defines the stable domain configuration. The film's D is the parameter for which the stable domain configuration has the measured scale (5 μm). **c** Virtual stripe domain pattern. The width matches that given by the domain average of **a**. **d** Same as **b** for pattern **c**. **e-h** Measured domain pattern for the perpendicular

demagnetized film and corresponding D-calculation and virtual stripe pattern calculation, following **a-d**. **i-l** additional patterns used for calculating D (calculation diagram not shown). The average from the measurements is indicated after the brace. **m-t** Same as **e-l** for in-plane demagnetized film A.

Film thickness variation from measured background MFM data.

MFM images contain noise, which on account of being random has no correlation when compared across different measurements. Features that are retained across a series of images are therefore due to reproducible interactions. Across all field levels it is evident from Figure 3 in the main text that the ± 0.5 Hz background pattern is essentially the same, proving it is not caused by instrument noise. The pattern could arise from topographical features (roughness) by van der Waals interaction between tip and sample (note that our non-contact measurement controls the electrostatic forces) or result from an inhomogeneous sample magnetic moment areal density, the stray field of which would interact with the tip magnetization. Note that in the former case the interaction is independent of the magnetization direction, whereas in the latter it changes sign with the relative sign of tip and sample magnetization. This distinguishing feature allows establishing the extent of either possible contribution.

To more clearly illustrate the method, it is convenient to work with larger domains, analogous to the case found in Joshi et al.[3] For such domains the main MFM contrast generated by the up/down domain pattern arises at the walls (domain wall contrast) such that contrast variations inside the domains become clearly visible. For illustration purposes we utilize the calibration sample B. We then measure the same area in negative (i.e. parallel to the dark domains) saturation (Figure 2b), in which the bright domains have reversed, whereupon the magnetic tip-sample interaction contributed by the bright domains reverses its sign. Note that van der Waals interactions would *not* reverse sign. Crucially, we employ a capacitive feedback method[4] to retain one and the same tip-sample distance in both measurements, 12.0 ± 0.5 nm. This allows a direct comparison of their signals. Since domains have vanished in saturation, computing the numerical pixel-wise difference of the two measurements (Figure 2c) retains the domains of Figure 2a but removes any sign-conserving background contributions, in this case van der Waals interactions but also magnetic interactions from the dark domains in Fig 2a, though not those from the bright domains. Conversely, computing the sum (Figure 2d) will double the sign-conserving background

contributions and remove the sign-reversing ones. As we can see from Figure 2c in the bright domains the background amplitude is about doubled, whereas in the dark domains it is largely removed. This proves the main cause for the background contrast is predominantly magnetic. It can be removed from a domain image like Figure 2a if the corresponding saturation image (Figure 2b) is reversed locally according to whether domain and tip magnetization are parallel or antiparallel (Figure 2e), prior to subtraction (Figure 2f).

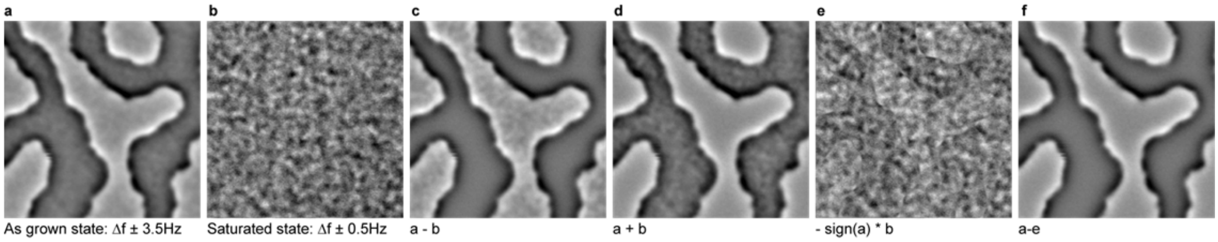

Figure 2: Background signal identification. 1 µm × 1 µm Δf images. **a** Domain state of the calibration sample after in-plane demagnetization in zero field. The magnetization of dark domains is parallel to the MFM tip (pointing in negative direction), and that of bright domains antiparallel. Away from the boundaries Δf is much smaller (in absolute terms) than at the boundaries (domain wall contrast), but different from zero and inhomogeneous. **b** Same area of **a** in saturation at −200mT (field parallel to the magnetization of the dark domains of **a**). Δf has average values comparable to those near the center of the domains in **a**, with peak inhomogeneity of ±0.5 Hz. **c** Pixel-by-pixel subtraction of image **b** from image **a**. Inside dark domains Δf is smaller and more homogeneous, but the opposite is true inside bright domains, suggesting the main part of Δf in **b** changes sign with relative tip-sample magnetization direction. **d** Pixel-by-pixel sum of image **b** and image **a**. Δf is smaller and more homogeneous inside the bright domains. **e** Image **b** multiplied pixel-wise with +1 over dark domains of **a** and -1 over bright domains of **a**. **f** Subtraction of image **e** from image **a** pixel by pixel. The background contrast inhomogeneity is removed from both domains confirming that the main part of Δf in **b** is of magnetic origin.

Local values of the DM interaction

A further example of the use of quantitative evaluation of high resolution images to determine the local value of D is given in Figure 3, which in essence repeats the analysis carried out in Figure 6 of the main text. It results in D = 3.470 ± 0.020 mJ/m^2 and 3.427 ± 0.020 mJ/m^2, respectively. Again, we observe that the difference between simulation and measurement is smallest along certain directions, here f and h for the top and bottom skyrmion, respectively. This supports the idea that the skyrmions are stabilized in a region of high D circumscribed to about 50 nm in diameter in which the thickness of the Co layers that constitute the multilayer is smaller than the average.

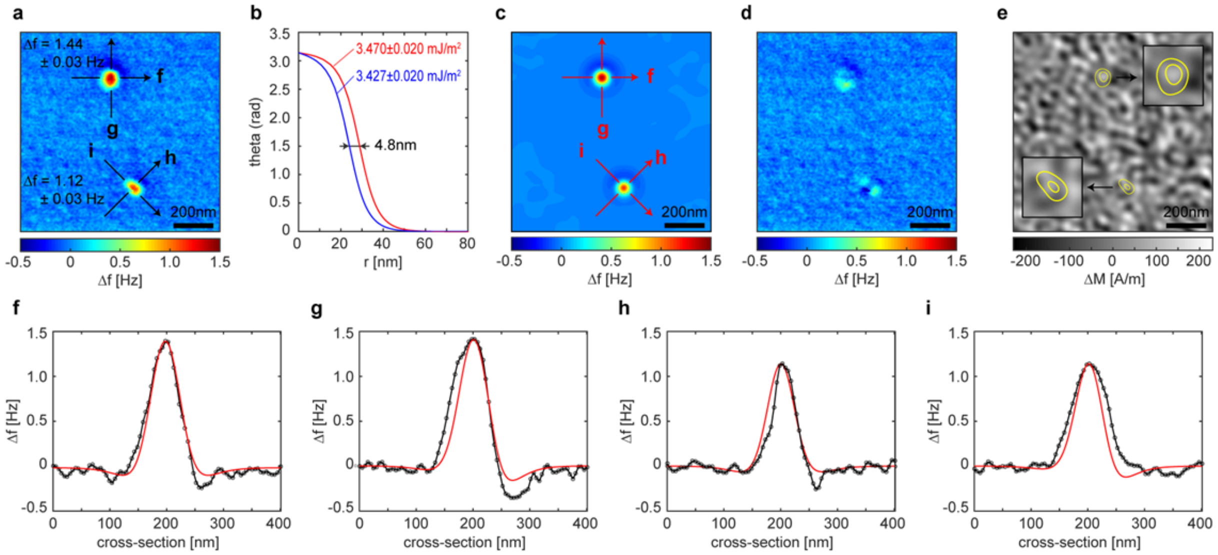

Figure 3: skyrmion measured and simulated profiles. **a** Background (i.e. same area in saturation) subtracted high resolution image of two skyrmions of sample A at 1.14 mT. The background has been subtracted. Sections f−i are reproduced in the corresponding black trace of Figures **f−i**. **b** Calculated skyrmion profiles for the values of D corresponding to the maximum contrast of top and bottom skyrmions. **c** Calculated measurement contrast maps for the skyrmion profiles of **b**. The sections f−i are reproduced in the corresponding red trace of Figures **f−i**. **d** Point by point map difference **a−c**. **e** Magnetization pattern which repeated on each Co layer reproduces the measured contrast in saturation, i.e. a measure of the local thickness' departure from the average 0.6 nm. **f−i** cross sections indicated in **a−c**.

Stability of the skyrmion solution used in modelling

In the main text we discussed the stability of a skyrmion solution that fitted the measurement. We studied it in terms of independent variations from the equilibrium of a single parameter (A, K_u, D). To complement this study, we show results of skyrmion simulations in which we allow also for variations of the areal density of the magnetization, M_{Co}.

Two types of variations are considered, resulting in the thick blue and maroon lines depicted in Figure 4. The thick blue lines in the figure represent points where stable skyrmion solutions were found for fixed $A = 16$ pJ/m, fixed K_u =414 kJ/m^3, and a grid of 11 × 101 values spanned by $M_{Co} = 457.52 - 784.32$ kA/m and $D = 2.98 - 3.51$ mJ/m^2 respectively. Similarly, the thick maroon lines denote the stable skyrmion solutions for a second set of parameters $A = 16$ pJ/m, and a grid of 11 × 151 values spanned by $M_{Co} = 367.7 -$

592.0 k A/m and $D = 3.3 - 4$ mJ/m^2 at constant $K_u M_{Co}$, hence $K_u = K_u^{avg} \cdot t^{avg}/_t$, while $M_{Co} = M_{Co}^{avg} \cdot {}^t/_{t^{avg}}$, where t is the local and t^{avg} the nominal film thickness. The simulated peak contrast for the stable skyrmions reaches the level found in experiment on the gray plane of the figure. Those points are joined by a thin blue or red line, respectively, to more clearly show the relation between the magnitude of D and M_{Co}. For independent variations of the parameters, we refer to the blue line and observe a small decrease of D as the M_{Co} decreases. This is qualitatively different from the behavior observed when $K_u M_{Co} = const$, as one could posit for the typical inverse film thickness dependence of the anisotropy (linear dependence for M_{Co}). However, in both cases we observe that as M_{Co} decreases a point is reached where the stable skyrmions do not reach the experimentally found peak contrast (see black arrows in the figure). The results suggest that although the local value of D could indeed vary with the type of codependence assumed for the parameters (an exercise we do not seek to address exhaustively here), it will remain significantly different (higher) than the average value.

Of course, the peak value of the contrast is not the complete measure of the fit between measurement and simulation. In fact, we calculate RMS value of the pixel-wise difference between simulation and experiment. The size of the circles in the projections of thin blue and red lines describes the relative variation (with an offset, for better view). Though qualitative in this form, the RMS difference is seen to decrease for decreasing M_{Co}, i.e. for decreasing local film thickness. The latter is compatible with increased values of local *D*.

A further point we would like to make is that analogous calculations we carried out for different values of A, from 10 to 18 pJ/m show the same qualitative behavior. We conclude that the finding of $D_{local} > D_{average}$ is robust.

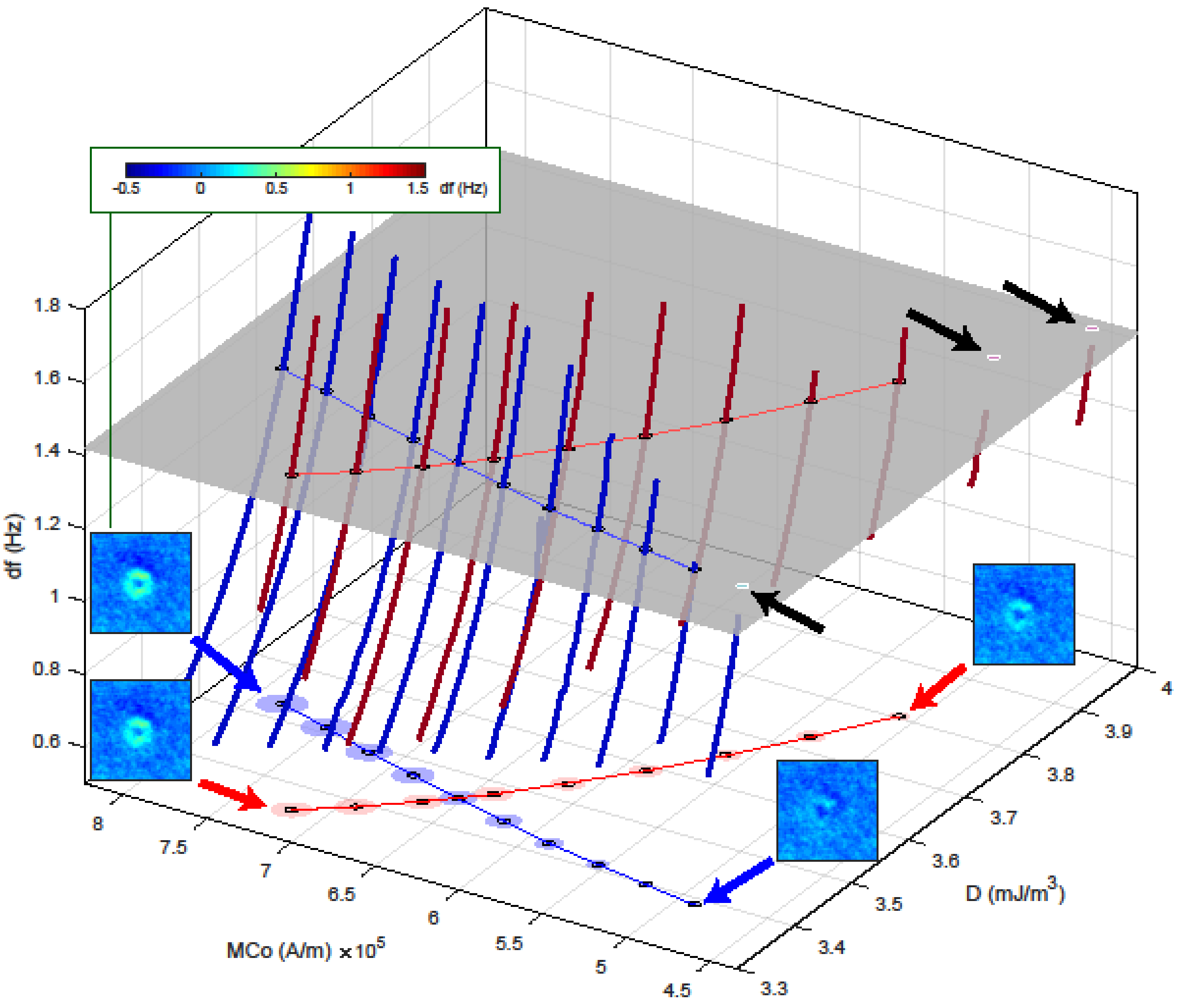

Figure 4: Peak skyrmion contrast from skyrmion simulations in the parameter space (A, K_u, M_{Co}, D). Shown is the peak skyrmion constrast on M_{Co} and D axes of the *stable* solutions from two sets of four-parameter points (A, K_u, M_{Co}, D), with a gray plane indicating the peak contrast of the skyrmion measured in Figure 3a,f,g. Set 1 comprises points of the form A = 16 pJ/m, K_u =414 kJ/m^3 , M_{Co} = 457.52 – 784.32 kA/m (11 values) and D = 2.98 – 3.51 mJ/m^2 (101 values), such that M_{Co} and D effectively vary independently. The set yields the thick blue lines that intersect the df = 1.42 Hz level at the thin blue line, also projected onto the $df = 0$ plane. Similarly, set 2 comprises points of the form A = 16 pJ/m, M_{Co} = 457.52 – 784.32 kA/m (11 values) and D = 3.3 – 4 mJ/m^2 (151 values), with K_u =367.7 – 592.0 kJ/m^3 such that $K_u M_{Co} = const$ (this amounts to inverse film thickness dependence for these parameters). This set yields the thick maroon lines that intersect the df = 1.42 Hz level at the thin red line, likewise projected to the bottom plane. Black arrows denote the absence of stable skyrmions with adequate peak contrast (on the gray plane) for the lowest magnetization values. For all simulations intersecting the gray plane, we compute the difference between the simulated skyrmion measurement and the measurement itself (examples denoted with red and blue arrows; image size 396 nm). The RMS difference over all image pixels in each case is measured by the diameter of the circle denoting each point on the projected lines.

References

[1] Moreau-Luchaire, C. et al. Additive interfacial chiral interaction in multilayers for stabilization of small individual skyrmions at room temperature. *Nature Nanotech.* ***11***, 444–448 (2016).

[2] Boulle, O. *et al.* Room-temperature chiral magnetic skyrmions in ultrathin magnetic nanostructures. *Nature Nanotech.* **11**, 449–454 (2016).

[3] Joshi, N. R. et al. Engineering the ferromagnetic domain size for optimized imaging of the pinned uncompensated spins in exchange-biased samples by magnetic force microscopy. *Appl. Phys. Lett*, **98**, 82502 (2011).

[4] Schwenk, J. et al. Bimodal magnetic force microscopy with capacitive tip-sample distance control. *Appl. Phys. Lett*. **107**, 132407 (2015).
